# Amyloid hypothesis: 20 years of failure in Alzheimer’s research

**DOI:** 10.1002/alz.095760

**Published:** 2025-01-09

**Authors:** Lloyd Tran, Zung Vu Tran

**Affiliations:** ^1^ Biomed Industries, Inc., San Jose, CA USA

## Abstract

**Background:**

We reviewed the data of past failures to determine how aducanumab and lecanemab might differ from the rest of this class of drugs. Specifically, whether the differences between treatment and placebo are different.

**Method:**

We systematically analyzed the data in publications where treatments were an anti‐Aβ monoclonal antibody drug, and data were available from published clinical trials. Only anti‐Aβ monoclonal antibody drugs that have completed at least a phase 2 trial were included. This class of drugs included, aducanumab, bapineuzumab, crenezumab, donanemab, gantenerumab, lecanemab, ponezumab, semorinemab, and solanezumab. We focused on two outcome measures that were most frequently reported ‐ CDR‐SB and ADAS‐Cog. Nearly all trials reported the post‐treatment difference between drug and placebo, i.e., the difference between scores of the treatment relative to the placebo at the end of the trial. On a scale where a higher score is “worse,” a negative (‐) difference “favors” treatment (lower score – higher score = ‐ value). We use bootstrapping methodology, a machine‐learning statistical procedure that resamples from a single dataset (with replacement) to create multiple samples. This resampling distribution allows for the calculation of means, median, standard errors, confidence intervals (CI), and other statistics. The means and 95%CI of these published differences were estimated by performing 10,000 resamples.

**Result:**

All the clinical trials analyzed were randomized, placebo controlled. Sample sizes varied for the phase 3 trials, from 253 to 1072 per arm (median = 519 participants); trial lengths were from 76 to 105 weeks (median = 78 weeks). The estimated bootstrap mean and 95%CI for differences between placebo and drug, for CDR‐SB and for ADAS‐Cog were: CDR‐SB; mean (95%CI) = *‐0.08* (‐0.31 to +0.22), range of differences = ‐0.70 to 1.30; ADAS‐Cog; mean (95%CI) = ‐*0.53* (‐1.03 to +0.22), range of differences = ‐1.60 to +4.74.

**Conclusion:**

The cumulative mean changes for CDR‐SB were ‐0.08; for ADAS‐Cog it was ‐0.53, with both 95%CI including zero. The score range for CDR‐SB is 0 to 18; the range for ADAS‐Cog is 0 to 70/90. These mean differences across the many trials conducted cannot be considered to be either clinically or statistically significant.

